# Characteristics of poorly controlled Type 2 diabetes patients in Swiss primary care

**DOI:** 10.1186/1475-2840-11-70

**Published:** 2012-06-15

**Authors:** Anja Frei, Stefanie Herzog, Katja Woitzek, Ulrike Held, Oliver Senn, Thomas Rosemann, Corinne Chmiel

**Affiliations:** 1 Institute of General Practice and Health Services Research, University Hospital of Zurich, Zurich, Switzerland; 2 Horten Centre for Patient-oriented Research, University Hospital of Zurich, Zurich, Switzerland

**Keywords:** Type 2 diabetes, General practitioner, Primary care, Guideline adherence, Determinants of HbA1c

## Abstract

**Background:**

Although a variety of treatment guidelines for Type 2 diabetes patients are available, a majority of patients does not achieve recommended targets. We aimed to characterise Type 2 diabetes patients from Swiss primary care who miss HbA1c treatment goals and to reveal factors associated with the poorly controlled HbA1c level.

**Methods:**

Cross-sectional study nested within the cluster randomised controlled Chronic Care for Diabetes study. Type 2 diabetes patients with at least one HbA1c measurement ≥7.0 % during the last year were recruited from Swiss primary care. Data assessment included diabetes specific and general clinical measures, treatment factors and patient reported outcomes.

**Results:**

326 Type 2 diabetes patients from 30 primary care practices with a mean age 67.1 ± 10.6 years participated in the study. The patients’ findings for HbA1c were 7.7 ± 1.3 %, for systolic blood pressure 139.1 ± 17.6 mmHg, for diastolic blood pressure 80.9 ± 10.5 mmHg and for low density lipoprotein 2.7 ± 1.1. 93.3 % of the patients suffered from at least one comorbidity and were treated with 4.8 ± 2.1 different drugs. No determining factor was significantly related to HbA1c in the multiple analysis, but a significant clustering effect of GPs on HbA1c could be found.

**Conclusions:**

Within our sample of patients with poorly controlled Type 2 diabetes, no “bullet points” could be pointed out which can be addressed easily by some kind of intervention. Especially within this subgroup of diabetes patients who would benefit the most from appropriate interventions to improve diabetes control, a complex interaction between diabetes control, comorbidities, GPs’ treatment and patients’ health behaviour seems to exist. So far this interaction is only poorly described and understood.

**Trial registration:**

Current Controlled Trials ISRCTN05947538.

## Background

Type 2 diabetes is one of the most common chronic diseases with an increasing prevalence worldwide [1]. A recently published study estimated the prevalence of Type 2 diabetes in Switzerland between 5.7 % and 7.0 % [2]. This chronic condition is a major challenge for the health care system and especially for primary care, where most of the patients are treated. In the long term, Type 2 diabetes results in serious vascular, nephrological, neurological and ophthalmological complications [3-5].

Today, a variety of treatment approaches, especially pharmaceutical drugs, are available which enable physicians to achieve the recommended treatment goals in nearly all patients [6,7]. But in reality, a majority of patients does not achieve recommended treatment targets, neither regarding the HbA1c level, nor the recommendations regarding blood pressure and lipid level. The so called “evidence-performance-gap”, describing the gap between evidence from large clinical trials and the daily performance in real life is remarkable, even if some recent studies suggested that a very low HbA1c level might not be beneficial for all patients. Undoubtedly, diabetic patients take the largest benefit from appropriate control of blood sugar, blood pressure and lipid level [8-12].

In this study we focused on primary care patients missing the actual HbA1c treatment targets. Our aim was to characterise these patients and to reveal possible factors associated with the poorly controlled HbA1c level. These factors could be possible targets in tailored interventions aiming at poorly controlled Type 2 diabetes patients.

## Methods

### Study design

The data on which this cross-sectional study is based were collected within the baseline assessment of patients participating in the cluster randomised controlled Chronic Care for Diabetes study (CARAT, ISRCTN05947538) [13]. According to a power calculation for cluster randomised trials, we planned to recruit 28 GPs who subsequently included 12 patients suffering from poorly controlled Type 2 diabetes. About 800 randomly selected GPs from the Eastern part of Switzerland were invited to an information meeting on the study. Additionally, the project was presented in several quality circle meetings in doctors’ networks. Detailed information on the study is given elsewhere [13]. The study protocol has been approved by the ethics committee of the Kanton Zurich and received an unrestricted positive vote on 25.01.2010.

### Patients

Eligible patients were identified through the GPs registry based on laboratory and received an initial letter by the GPs containing information on the study. All patients were included in consecutive order of appearance in the practice, regardless the reason for the current encounter. The inclusion criteria to participate in the study were adulthood, a diabetes mellitus Type 2 diagnosis (initially diagnosed with a Fasting Glucose in blood plasma >7.0 mmol / 126 mg/dl) and at least one HbA1c measurement ≥7.0 % during the last year. The latter criterion was formulated because the aim of CARAT was to reduce the primary outcome HbA1c with the intervention by 0.5 % points considering the current recommendations (HbA1c = 6.5 %) at study onset [7]. Exclusion criteria were insufficient language skills to read and understand informed consent, patient information and the questionnaires, practice contact for emergencies only (e.g. no continuous patient-doctor relationship) and a life expectancy less than six months due to oncological or other severe diseases.

### Measures and data collection

After the patients gave their written informed consent, a questionnaire was filled out by the GPs and the practice nurses for each participant containing questions on diagnostic findings, comorbidities and diabetes associated complications (retinopathy, nephropathy and peripheral vascular disease), current medication, number of consultations during the past year and an estimation regarding the patient’s compliance using a 4-point scale (1 = very good, 4 = very bad, used previously [14]). Current clinical and laboratory measures such as HbA1c, blood pressure, fasting blood glucose, body weight, LDL, HDL, total cholesterol and pulse were assessed by the practice team using point-of-care laboratory analysis and/or external laboratories. Creatinine clearance was calculated by simplified MDRD and Cockcroft formula.

A second questionnaire was filled out by the patients containing questions about patients’ characteristics, smoking behaviour, Patient Health Questionnaire short form PHQ-9 [15], Medical Outcomes Study 36-Item Short-Form Survey (SF-36) [16] and Patient Assessment of Chronic Illness care (PACIC) [17,18].

The PHQ-9 [15] contains 9 items which correspond to the DSM-IV criteria for major depression. The items are scored using a Likert Type scale ranging from 0 (=not at all) to 3 (=almost every day). A total depressive summary score is constructed, and the following interpretation is suggested by the authors: 0-4 = no, 5-9 = light, 10-14 = moderate, 15-19 = severe, 20-27 = most severe depressive disorder with a recommended cut-off score of 10 points for major depression. The PHQ-9 has been proven to be a valid and reliable tool to assess depression, has already been used in previous studies in primary care [19,20] and was recently validated in diabetes patients [21].

The generic quality of life instrument SF-36 assesses physical and mental functioning and comprises the eight domains physical functioning, physical role (role limitation due to physical problems), bodily pain, general health, vitality, social functioning, emotional role (role limitation due to emotional problems) and mental health. For all these multi-item domains, the raw summary scores were transformed into a 0 to 100 scale (higher scores indicate better health status) according to the SF-36 manual [22]. The physical component summary scale (PCS) and mental component summary scale (MCS) were calculated by considering the mean, SD and regression coefficients from the proposed American norm population of the SF-36 manual [22].

The PACIC has been developed to assess congruency of provided health care to the Chronic Care Model (CCM) [23-26]. It is organized according to the key elements of the CCM and assesses the behaviour of professionals and practice teams from a patient’s perspective. It contains 20 items which reflect the 5 scales patient activation, delivery system design/decision support, goal setting/tailoring, problem solving/contextual and follow-up/coordination. The items are scored on a 5-point Likert scale, ranging from 1 (almost never) to 5 (almost always). Recently, a German version of the PACIC has been validated in a sample of osteoarthritis patients [18].

The GPs created a list with the participants and allocated a code to each patient. Both questionnaires were marked with this patient code and sent to the University in a stamped envelope independently by the patients and practice teams. The University had no access to the patients’ names, anonymity was therefore ensured.

### Statistical analysis

Continuous variables are presented as means and standard deviations (SD), categorical data as frequencies and percentages. Bivariate association measurements between the HbA1c and continuous variables were conducted using Pearson correlations, between HbA1c and categorical variables using t-tests and one-way ANOVA (more than two groups). Multiple regression analysis was applied to examine the independent association between HbA1c and patient characteristics. We included all variables in the regression model that showed a significant relationship with HbA1c on a 10 % level in the bivariate analyses and, in addition, could be interpreted as potential determinants of HbA1c in terms of content. We supplemented the model with potential determinants of HbA1c according to the literature which were not significant in the bivariate analyses. We further used multilevel regression analysis with the GP as cluster level, thus taking into account that patient observations are not independent, i.e. observations in one cluster tend to be more similar to each other than to individuals in the rest of the sample. The amount of clustering (i.e. the variation that is explained by the GP level) was assessed by calculating the intraclass correlation coefficient (ICC) ranging from 0 % (i.e. no evidence for clustering) to 100 % (i.e. all the variation in HbA1c difference is explained on GP level). A two-sided alpha of 0.05 was set as level of significance for all comparisons. All analyses were calculated using STATA statistical package, version 11.2 (Stata Incorporation, College Station, TX, USA) or SPSS Statistics 19.0 software.

## Results

### GP characteristics

30 GPs from the German speaking part of Switzerland participated in the study. Their mean age was 50.7 ± 7.2 years and 27 (90 %) were male. 10 (33.3 %) GPs worked in single handed and 20 (66.7 %) in group practices. 17 (56.7 %) were member of a doctor’s network (“Aerztenetz”) whereas 13 (43.3 %) were not.

### Patient and treatment characteristics of the sample

#### Patient characteristics and clinical measures

Between February and May 2010 326 patients (7–13 per practice) with poorly controlled Type 2 diabetes were enrolled into the study. 57.4 % were male with a mean age of 67.1 ± 10.6 years. 11.5 % currently smoked and 41.1 % were former smokers. The majority (57.6 %) had a positive family history of Type 2 diabetes (1^st^ grade) and their diabetes has been diagnosed 9.9 ± 7.6 years ago on average. The mean HbA1c level was 7.7 ± 1.3 %, the mean systolic blood pressure 139.1 ± 17.6 mmHg, the mean diastolic blood pressure 80.9 ± 10.5 mmHg and the mean low density lipoprotein (LDL) 2.7 ± 1.1. On average, patients had eight consultations at the GPs in the past year and their compliance was mostly rated by the GPs as “good” (46.0 %) and “very good” (33.6 %) (Table 1). 16.9 % of the patients achieved the recommended targets [7] regarding blood pressure (<130/80), 49.5 % regarding LDL-cholesterol (<2.6 mmol/l).

**Table 1:** Patient characteristics, clinical measures and association with HbA1c

	**Mean ± SD or n (%)**	**Association with HbA1c**^**1)**^	
		**Coefficient**	***P*-value**
**Anthropometrics and sociodemographics**			
Age (years)	67.1 ± 10.6	-0.167	0.003 ^a)^
Male gender (n, %)	187 (57.4)		0.675 ^b)^
Nationality Swiss (n, %)	291 (91.8)		0.228 ^b)^
Living together with partner/family (n, %)	246 (78.3)		0.392 ^b)^
Still working (n, %)	100 (32.2)		0.249 ^b)^
Education (years)	11.6 ± 3.2	-0.027	0.632 ^a)^
Smoking status			0.780 ^c)^
Current smoker (n, %)	36 (11.5)		
Former smoker (n, %)	129 (41.1)		
Never smoker (n, %)	149 (47.4)		
**Diabetes variables**			
Duration of diabetes (years)	9.9 ± 7.6	0.019	0.740 ^a)^
Family history of Type 2 diabetes (n, %)	186 (57.6)		0.987 ^b)^
Glycated haemoglobin (HbA1c, %)	7.7 ± 1.3		
Fasting serum glucose (mmol/l)	8.0 ± 2.4	0.472	0.000 ^a)^
Severe hypoglycaemia (≥1, %) ^2)^	32 (9.8)		0.937 ^b)^
Hyperglycaemic episodes (≥1, %) ^2)^	19 (5.8)		0.091 ^b), 4d)^
Self monitoring of blood glucose			
none (n, %)	65 (19.9)		0.352 ^b)^
daily (≥1) (n, %)	133 (40.8)		0.122 ^b)^
weekly (≥1) (n, %)	101 (31.0)		0.205 ^b)^
monthly (≥1) (n, %)	27 (8.3)		0.707 ^b)^
Foot status (pathological; n, %)	52 (16)		0.105 ^b)^
Peripheral pulse status (pathological; n, %)	97 (29.8)		0.387 ^b)^
Monofilament test (pathological; n, %)	42 (13.5)		0.091 ^b), 4e)^
Vibratory sensation (pathological; n, %)	81 (24.9)		0.735 ^b)^
Annual dilated eye exam			
pathological (n, %)	28 (8.7)		0.898 ^b)^
non-pathological (n, %)	194 (60.1)		0.376 ^b)^
not conducted (n, %)	101 (31.2)		0.372 ^b)^
**Clinical and laboratory measures**			
Number of consultations last year	8.07 ± 6.0	-0.051	0.355 ^a)^
Compliance (1 = very good, 4 = very bad)	1.87 ± 0.7	0.161	0.004 ^a)^
			0.027 ^c),3),4f)^
very good	109 (33.6)		
rather good	149 (46)		
rather bad	64 (19.8)		
very bad	2 (0.6)		
Blood pressure systolic (mmHg)	139.1 ± 17.6	-0.004	0.941 ^a)^
Blood pressure diastolic (mmHg)	80.9 ± 10.5	0.101	0.070 ^a)^
Pulse	73.7 ± 11.9	0.024	0.671 ^a)^
Body-mass index (BMI; kg/m^2^)	30.6 ± 5.6	0.137	0.013 ^a)^
Waist circumference (cm)	108.3 ± 12.4	0.105	0.059 ^a)^
Waist-hip ratio (cm/cm)	1.0 ± 0.1	0.006	0.910 ^a)^
Cholesterol (mmol/l)			
Total	4.8 ± 1.1	0.069	0.214 ^a)^
Low-density lipoprotein (LDL)	2.7 ± 1.1	0.072	0.196 ^a)^
High-density lipoprotein (HDL)	1.2 ± 0.4	-0.088	0.113 ^a)^
Creatinine (μmol/l)	82.1 ± 26.8	-0.009	0.866 ^a)^
Creatinine-Clearance according to Cockcroft	96.3 ± 47.6	0.108	0.052 ^a)^

^1) a)^ Associations HbA1c with continuous variables by Pearson correlations (coefficient), between HbA1c and categorical variables by ^b)^ t-tests and ^c)^ ANOVA.

^2)^ In the last 12 months.

^3)^ Compliance categories “rather bad & very bad” categorised because of “very bad” n = 2.

^4)^ HbA1c (%) mean per category of significant t-test and ANOVA results (p < 0.010): ^d)^ Hyperglycaemic episodes: yes = 8.5, no = 7.6; ^e)^ Monofilament test: pathological = 7.5, non-pathological = 7.8; ^f)^ Compliance: very good = 7.5, rather good = 7.7, rather bad & very bad = 8.0.

#### Comorbidities, diabetes associated complications and medication

93.3 % of the patients suffered from at least one comorbidity, more than half of the patients had three or more comorbidities. The most frequent comorbidity was arterial hypertension (71.3 %), followed by hyperlipidemia (66.7 %) and adipositas (53.1 %) (Table 2). Diabetes associated microvascular complications were diagnosed in 8.6 % of the patients; most frequently diabetic retinopathy (5.3 %), followed by peripheral vascular disease (3.1 %) and diabetic nephropathy (1.8 %).

**Table 2:** Comorbidities and association with HbA1c

	**Mean ± SD or n (%)**	**Association with HbA1c**^**1)**^	
		**Coefficient**	***P*-value**
Number of comorbidities	2.66 ± 1.6	-0.041	0.459 ^a)^
≥1 comorbidity (n, %)	304 (93.3)		0.297 ^b)^
0 comorbidities (n, %)	22 (6.7)		
1 comorbidity (n, %)	59 (18.1)		
2 comorbidities (n, %)	73 (22.4)		
3 comorbidities (n, %)	86 (26.4)		
≥4 comorbidities (n, %)	86 (26.4)		
Hypertension (n, %)	231 (71.3)		0.049 ^b), 4c)^
Hyperlipidemia (n, %)	208 (66.7)		0.097 ^b), 4d)^
Adipositas (n, %)^2)^	173 (53.1)		0.057 ^b), 4e)^
Obese class I (30 – 34.9) (n, %)	111 (34.1)		
Obese class II (35 – 39.9) (n, %)	42 (12.9)		
Obese class III (≥ 40) (n, %)	20 (6.1)		
Coronary heart disease (n, %)	66 (20.4)		0.350 ^b)^
Depression (n, %)	38 (11.7)		0.855 ^b)^
Asthma / COPD (n, %)	32 (9.8)		0.460 ^b)^
Myocardial infarction (n, %)^3)^	30 (9.3)		0.530 ^b)^
Heart failure (n, %)	22 (6.8)		0.823 ^b)^
Stroke (n, %)^3)^	17 (5.2)		0.747 ^b)^
Cancer (n, %)^3)^	13 (4.2)		0.570 ^b)^

^1) a)^ Associations HbA1c with continuous variables by Pearson correlations, ^b)^ between HbA1c and categorical variables by t-tests.

^2)^ BMI ≥ 30.

^3)^ Present or history of.

^4)^ HbA1c (%) mean per category of significant t-test and ANOVA results (p < 0.010): ^c)^ Hypertension: yes = 7.6, no = 8.0; ^d)^ Hyperlipidemia: yes = 7.6, no = 7.8; ^e)^ Adipositas: yes = 7.8, no = 7.6.

On average, patients were treated with 4.8 different drugs. Almost all (96.6 %) received an antidiabetic medical therapy; more frequently an oral medication (88.2 %) than insulin (31.8 %), in 23.8 % a combination of the two. Additionally, 76.2 % of the patients received an antihypertensive agent, 55.4 % antiplatelet therapy, 56.3 % lipid-lowering therapy and 11.5 % antidepressants (Table 3).

**Table 3:** Medication and association with HbA1c

	**Mean ± SD or n (%)**	**Association with HbA1c**^**1)**^	
		**Coefficient**	***P*-value**
Anitdiabetic therapy	312 (96.6)		0.022 ^c), 3d)^
None (n, %)	12 (3.7)		
Only oral (n, %) ^2)^	208 (64.4)		
Only insulin (n, %) ^2)^	26 (8.0)		
Combined (insulin and oral) (n, %) ^2)^	77 (23.8)		
Specific antidiabetic therapies			
Insulin (n, %)	103 (31.9)		0.005 ^b), 3e)^
Metformin/Biguanid (%)	254 (78.6)		0.384 ^b)^
Sulfonylurea (n, %)	136 (42.1)		0.764 ^b)^
Gliptine (DPP-III) (n, %)	44 (13.6)		0.425 ^b)^
Glitazone (n, %)	19 (5.9)		0.553 ^b)^
Glinide (n, %)	10 (3.1)		0.160 ^b)^
Incretin mimetic (n, %)	6 (1.9)		0.090 ^b) 3f)^
α-Glucosidase inhibitor (n, %)	4 (1.2)		0.837 ^b)^
Any antihypertensive agent (n, %) ^2)^	246 (76.2)		0.098 ^b), 3g)^
Diuretics (n, %)	160 (49.5)		0.317 ^b)^
Inhibitor of the angiotensin converting enzyme (ACE-I) (n, %)	156 (48.3)		0.213 ^b)^
Beta-blocker (n, %)	101 (31.3)		0.321 ^b)^
Angiotensin II inhibitor (ARB) (n, %)	70 (21.7)		0.787 ^b)^
Calcium antagonists (n, %)	64 (19.8)		0.135 ^b)^
others (n, %)	4 (1.2)		0.838 ^b)^
Any antiplatelet therapy (n, %) ^2)^	179 (55.4)		0.700 ^b)^
Aspirin (n, %)	158 (48.9)		0.869 ^b)^
Phenprocoumon (n, %)	21 (6.5)		0.017 ^b), 3h)^
Clopidogrel (n, %)	17 (5.3)		0.505 ^b)^
Any lipid-lowering therapy (n, %) ^2)^	182 (56.3)		0.005 ^b), 3i)^
Any antidepressants (n, %)	37 (11.5)		0.751 ^b)^
Number of drugs total	4.8 (2.1)	-0.052	0.349 ^a)^

^1) a)^ Associations HbA1c with continuous variables by Pearson correlations, between HbA1c and categorical variables by ^b)^ t-tests and ^c)^ ANOVA.

^2)^ 1 or more drugs possible.

^3)^ HbA1c (%) mean per category of significant t-test and ANOVA results (p < 0.010): ^d)^ Anitdiabetic therapy: none = 7.2, only oral = 7.6, only insulin = 7.8, combined (insulin and oral) = 8.0; ^e)^ Insulin: yes = 8.0, no = 7.6; ^f)^ Incretin mimetic: yes = 8.6, no = 7.7; ^g)^ Any antihypertensive agent: yes = 7.6, no = 8.0; ^h)^ Phenprocoumon: yes = 7.4, no = 7.7; ^i)^ Any lipid-lowering therapy: yes = 7.5, no = 7.9.

#### Patient reported outcomes

Means and SD of SF-36, PACIC and PHQ-9 are presented in Table 4. According to the PHQ-9, 16.2 % of the patients met cut-off criteria for major depression.

**Table 4:** Patient reported outcomes (SF-36, PACIC and PHQ-9) and association with HbA1c

	**Mean ± SD or n (%)**	**Association with HbA1c**^**1)**^	
		**Coefficient**	***P*-value**
**SF-36**			
Physical functioning	71.3 ± 26.2	0.003	0.961 ^a)^
Physical role	64.8 ± 41.7	-0.056	0.326 ^a)^
Bodily pain	67.8 ± 29.7	-0.006	0.921 ^a)^
General health	61.0 ± 18.1	-0.063	0.271 ^a)^
Vitality	57.3 ± 21.5	-0.105	0.065 ^a)^
Social functioning	79.7 ± 23.8	-0.040	0.483 ^a)^
Emotional role	75.6 ± 39.2	-0.068	0.231 ^a)^
Mental health	73.1 ± 19.7	-0.055	0.337 ^a)^
Physical component summary scale (PCS)	43.9 ± 10.9	-0.020	0.723 ^a)^
Mental component summary scale (MCS)	50.1 ± 11.3	-0.090	0.117 ^a)^
**PACIC** summary score	3.18 ± 0.85	-0.041	0.491 ^a)^
Patient activation	3.83 ± 1.13	-0.037	0.520 ^a)^
Delivery system/practice design	3.87 ± 0.82	-0.019	0.749 ^a)^
Goal setting/tailoring	2.86 ± 0.98	-0.066	0.262 ^a)^
Problem solving/contextual	3.26 ± 1.22	-0.060	0.306 ^a)^
Follow-up/coordination	2.66 ± 1.05	0.031	0.605 ^a)^
**PHQ-9** summary score	5.21 ± 4.76	0.043	0.457 ^a)^
Major Depression overall (10-27)	49 (16.2%)		0.705 ^b)^
No depressive disorder (0-4)	169 (56%)		0.255 ^b)^
Light depressive disorder (5-9)	84 (27.8%)		0.342 ^b)^
Moderate depressive disorder (10-14)	35 (11.6%)		0.953 ^b)^
Severe depressive disorder (15-19)	8 (2.6%)		0.525 ^b)^
Most severe depressive disorder (20-27)	6 (2%)		0.895 ^b)^

^1) a)^ Associations HbA1c with continuous variables by Pearson correlations, ^b)^ between HbA1c and categorical variables by t-tests.

### Associations with HbA1c

In the bivariate analysis, following factors which fulfilled our criteria for the regression model were significantly associated with a higher HbA1c (Tables 1, 2, 3 and 4): age (younger), compliance (lower), antidiabetic therapy (none: lowest, with insulin higher, combined highest), BMI (higher) and SF-36 domain vitality (lower). Except for antidiabetic therapy, we did not include any pharmacological treatment into the multiple model. Hyperglycaemic episodes, monofilament test and creatinine-clearance were also not included as they were judged to be results of HbA1c instead of possible determinants. Variables not included due to colinearity with model variables were fasting serum glucose, therapy with insulin, waist circumference and adipositas. Hypertension, hyperlipidemia and diastolic blood pressure were not considered because they were represented by the included variable number of comorbidities (see below). Based on literature review, we additionally included sex, duration of diabetes (supposed: longer), living together with partner/family (supposed: no), smoking status (supposed: current smoking), depression (PHQ summary score, supposed: higher) and number of comorbidities (supposed: more). The results of the multiple linear regression are presented in Table 5. No significant correlation between the investigated determinants and HbA1c could be found (R squared 0.09). When controlled for clustering effect of GPs, age achieved a significant small inverse association with HbA1c in the multilevel regression analysis (coefficient -.019, p = 0.033). The clustering effect of GPs on HbA1c was significant (ICC = 12.9 %, p = 0.001).

**Table 5:** Influence on HbA1c: Multiple regression analysis

**Independent variables**	**Coefficient**	** 95% CI**	***P*-value**
Age (years)	-0.016	-0.033 – 0.002	0.085
Sex	0.055	-0.272 – 0.381	0.744
Compliance			
good (ref = very good)	0.237	-0.110 –0.584	0.180
bad & very bad (ref = very good)^1)^	0.304	-0.138 –0.746	0.178
Antidiabetic therapy			
only oral (ref = none)	0.258	-0.628 – 1.144	0.569
only insulin (ref = none)	0.469	-0.589 – 1.526	0.385
combined (ref = none)	0.728	-0.210 – 1.666	0.128
BMI	0.030	-0.005 – 0.064	0.093
Vitality (SF-36 subscale)	-0.010	-0.020 – 0.001	0.069
Duration of diabetes	0.008	-0.016 – 0.032	0.518
Living together with partner/family	0.139	-0.243 – 0.520	0.477
Smoking			
smoker (ref = never smoker)	0.028	-0.494 – 0.549	0.917
ex-smoker (ref = never smoker)	0.087	-0.254 – 0.428	0.618
Depression (PHQ-9)	-0.032	-0.080 – 0.015	0.182
No of comorbidities	-0.120	-0.241 – 0.001	0.052

^1)^ Rather bad & very bad categories are merged to one category.

## Discussion

Type 2 diabetes patients with an HbA1c above 7 % treated in Swiss primary care are multimorbid and do not achieve current guideline targets regarding cardiovascular risk factors blood pressure and LDL-cholesterol. In contrast to our assumption, the regression analysis did not reveal significant key factors which determine the HbA1c level. A cluster effect of GPs on HbA1c could be found.

When comparing the demographics of our study population to recently published results of treated Type 2 diabetes primary care patients in Europe [27,28] and specifically in Switzerland [29], the mean age, slightly higher male percentage, time since diabetes diagnosis and frequency of diabetes family history were comparable. The higher HbA1c of the patients in our sample is caused by inclusion criteria (at least one HbA1c measurement ≥7 % during the last year). In terms of cardiovascular risk factors, more patients achieved the recommended targets [7] regarding LDL-cholesterol (<2.6 mmol/l) and slightly less regarding blood pressure (<130/80) as in the mentioned recent studies [27,28]. This is remarkable since only patients with insufficiently controlled diabetes were included in our study and it could indicate that GPs focus more on the other cardiovascular risk factors when the diabetes is poorly controlled. Interestingly, smoking was less prevalent in our sample than in the Swiss general population [30], but 41 % were former smokers. More than half of the patients were obese, being in line with the results of other studies [28,29,31].

Our patient population was multimorbid, over 90 % had at least one, 75 % had at least two and more than half of the patients had three or more comorbid diseases. Multimorbidity of the study sample resulted in polypharmacy, on average each patient received almost 5 different drugs. Compared to a Spanish primary care sample [27], the Swiss patients were more frequently treated with antihypertensive, antiplatelet and lipid-lowering drugs. Microvascular complications overall, in particular retinopathy and nephropathy, were much less prevalent in our patients compared to the Spanish sample [27]. Interestingly, patients’ compliance rated by the GPs was mainly classified as very good or good, showing better ratings compared to results from German primary care [14]. Even though 16.2 % of the patients met criteria for major depression according to the PHQ-9 cut-off score, the PHQ-9 summary score in our sample was slightly lower than in another recently published study including diabetes patients [21].

Within our sample of insufficiently controlled diabetes patients it was not possible to clearly identify factors associated with HbA1c to reveal possible targets for interventions. Although compliance did not reveal a significant association in the multiple analysis, the bivariate associations of compliance and the SF-36 subscale vitality with HbA1c are in line with the literature [14,32,33]. The multiple regression model could only explain 9 % of the overall variation in HbA1c, moreover, no factors with a relevant or significant contribution could be detected. This is in contrast to previous studies which found e.g. depression to be significantly associated with higher HbA1c [32,33]. Reasons for these differences may be related to our focus on patients with poorly controlled diabetes and may also reflect that within this subgroup of patients, diabetes control is an even more complex issue with no simple “key targets” to address. The fact that many of the patients in our sample have already quit smoking may reflect their contribution to cardiovascular risk factor control. Since GPs seem already to focus on treatment of the other relevant cardiovascular risk factors blood pressure and LDL-cholesterol, weight control remains as only reasonable additional intervention. Altogether, these results may reflect a complex and so far unexplained interaction within this specific subgroup between diabetes, comorbidities, GPs’ treatment and patients’ health behaviour.

An interesting finding is the fact that a significant cluster effect occurred, which was higher than in previous studies [34]. In a recent study of our research group, investigating hypertension treatment in primary care (Chmiel et al., 2011, submitted), these cluster effects could not be identified, indicating that there is more standardisation in hypertension treatment than in diabetes control among GPs.

Since participation in this study was voluntary for all GPs, some selection bias has to be acknowledged. It is likely that the sample included merely GPs who were very interested in the topic diabetes. Furthermore, we only considered practices in the German part of Switzerland; hence regional differences are not considered [29]. Compared to the Swiss GP population statistics, the GPs participating in our study worked more frequently in group than single handed practices and were more frequently members of a doctor’s network [35]. Since the aim of the study was to focus on patients with not optimally controlled diabetes, the results cannot be transferred to all diabetes patients in primary care. Despite this selection, the characteristics of our sample were comparable to other European primary care diabetes Type 2 samples.

Since we did not restrict the inclusion criteria any further, a strength of the study is that we included a wide spectrum of patients presumably reflecting the “real life” situation of poorly controlled diabetes patients. In addition, we were able to collect numerous variables of patients’ characteristics, clinical data and patient reported outcomes leading to a comprehensive impression of this subsample of diabetes Type 2 patients in Switzerland.

## Conclusions

Within our sample of patients with poorly controlled diabetes, no “bullet points” which can be addressed easily by some kind of intervention could be pointed out. Especially within this subgroup of diabetes patients who would benefit the most from appropriate interventions to improve diabetes control, a complex interaction between diabetes control, comorbidities, GPs’ treatment and patients’ health behaviour seems to exist. So far this interaction is only poorly described and understood. More research is needed to define these interactions within this specific patient group more accurately to tailor successful interventions.

## Abbreviations

HbA1c, Glycated haemoglobin; BMI, Body mass index; LDL, Low density lipoprotein; HDL, High density lipoprotein.

## Competing interests

The authors declare that they have no competing interests.

## Authors’ contributions

TR, AF and CC were the initiators of the study and participated together with OS in the design of the study. AF and TR organised the recruitment of the practices. AF, CC and TR developed the questionnaires. SH and KW organised the data collection and management. UH and OS supervised the analyses and gave statistical and methodical input. AF, CC and SH performed the statistical analysis. AF, CC and OS drafted the report which the paper is based on. All authors contributed in writing and revising the manuscript. All authors read and approved the final manuscript.
